# Association of Time-of-Day Energy Intake Patterns with Nutrient Intakes, Diet Quality, and Insulin Resistance

**DOI:** 10.3390/nu13030725

**Published:** 2021-02-25

**Authors:** Xiaoyun Song, Huijun Wang, Chang Su, Zhihong Wang, Feifei Huang, Jiguo Zhang, Wenwen Du, Xiaofang Jia, Hongru Jiang, Yifei Ouyang, Yun Wang, Li Li, Gangqiang Ding, Bing Zhang

**Affiliations:** Department of Public Nutrition and Policy Standard, National Institute for Nutrition and Health, Chinese Center for Disease Control and Prevention, Beijing 100050, China; sxydljk@126.com (X.S.); wanghj@ninh.chinacdc.cn (H.W.); suchang@ninh.chinacdc.cn (C.S.); wangzh@ninh.chinacdc.cn (Z.W.); huangff@ninh.chinacdc.cn (F.H.); zhangjg@ninh.chinacdc.cn (J.Z.); Duww@ninh.chinacdc.cn (W.D.); jiaxf@ninh.chinacdc.cn (X.J.); jianghr@ninh.chinacdc.cn (H.J.); ouyyf@ninh.chinacdc.cn (Y.O.); wangyun@ninh.chinacdc.cn (Y.W.); lili@ninh.chinacdc.cn (L.L.); dinggq@chinacdc.cn (G.D.)

**Keywords:** energy intake, latent class analysis, diet quality, insulin resistance

## Abstract

Evidence shows time-of-day of energy intake are associated with health outcomes; however, studies of time-of-day energy patterns and their health implication are still lacking in the Asian population. This study aims to examine the time-of-day energy intake pattern of Chinese adults and to examine its associations with nutrient intakes, diet quality, and insulin resistance. Dietary data from three 24-h recalls collected during the 2015 China Health and Nutrition Survey (CHNS) were analyzed (*n* = 8726, aged ≥ 18 years). Time-of-day energy intake patterns were determined by latent class analysis (LCA). General Linear Models and Multilevel Mixed-effects Logistic Regression Models were applied to investigate the associations between latent time-of-day energy intake patterns, energy-adjusted nutrient intakes, diet quality score, and insulin resistance. Three time-of-day energy intake patterns were identified. Participants in the “Evening dominant pattern” were younger, had higher proportions of alcohol drinkers and current smokers. The “Evening dominant pattern” was associated with higher daily energy intake and a higher percentage of energy from fat (%) (*p* < 0.001), as well as higher insulin resistance risk (OR = 1.21; 95% CI: 1.05, 1.40), after adjusting for multivariate covariates. The highest diet quality score was observed in participants with “Noon dominant pattern” (*p* < 0.001). A higher proportion of energy in the later of the day was associated with insulin resistance in free-living individuals.

## 1. Introduction

There is a gradual trend in recent years toward meal timing to understand the relationships between diet and health outcomes. It is well known that the circadian rhythms of the human body generated by the central clock located in the suprachiasmatic nucleus regulate several physiological responses, including food absorption and nutrition metabolism. Besides, food intake also fine-tunes local peripheral clocks located in the gut, liver, and pancreas, etc. [1,2,3]. A mismatch between the circadian timing system and food intake might contribute to poor cardiometabolic health [4].

Epidemiologic evidence suggests that breakfast skipping [5,6], late dinner [7,8], and high energy intake at night [9,10] have been linked to various indicators of cardiometabolic diseases. However, most studies focused on isolated eating occasions, rather than the full spectrum of eating occasions, or the time-of-day or temporal eating pattern. Accordingly, recent analyses of the Australian National Nutrition and Physical Activity Survey (NNPAS) identified distinct temporal eating patterns by latent class analysis [3] and further examined the associations of latent temporal eating patterns with nutrient intake, diet quality, and measures of adiposity [11] and hypertension [12]. These studies provide novel approaches to assess variation in the timing of eating occasions over the day and their potential association with cardiometabolic factors. Yet, the question of whether there is a specific time-of-day pattern of energy distribution in a day that is more beneficial or detrimental to health remains unclear. Although studies [2,13,14] applying kernel k-Means Clustering with an appropriate distance metric to NHANES data based on energy contribution, time of dietary intake, and a number of intake occasions have identified different temporal dietary patterns in the adult U.S. population 20 years and older, the cluster techniques used in this study allocated subjects to clusters based on pre-determined cluster number and no statistical tests were available in determining the optimal number of clusters. Moreover, evidence regarding time-of-day energy intake patterns and their dietary profiles and health implications is still lacking in the Asian population.

Insulin resistance is a major contributing factor in the pathogenesis of type 2 diabetes mellitus (T2DM). Besides traditional approaches in the prevention and treatment of insulin resistance, such as physical activity, food intake, and medication, circadian factors including meal timing also regulate insulin secretion [15]. Evidence from a randomized crossover trial [16] showed that circadian misalignment caused by shift work led to insulin resistance in skeletal muscle. Observational studies showed that greater energy intake and carbohydrate consumption earlier in the day were associated with higher insulin sensitivity in individuals without diabetes [17]. However, it is unknown whether a specific time-of-day energy intake pattern is related to insulin resistance in a free-living and healthy population.

This study aimed to (1) examine the time-of-day energy intake patterns in the Chinese adult population; (2) to evaluate the sociodemographic and eating pattern profile by time-of-day energy intake patterns; (3) to evaluate the nutrient intakes and diet quality by time-of-day energy intake patterns; and (4) to examine the association between time-of-day energy intake patterns and insulin resistance.

## 2. Materials and Methods

### 2.1. Study Population

The present study used data from the China Health and Nutrition Survey (CHNS). Initiating in 1989, CHNS is an ongoing multipurpose longitudinal survey with the aim to capture social, economic, and demographic changes that occurred in China, and to unfold how these social and economic changes in China affect nutrition status and health-related outcomes across the life cycle [18]. The CHNS has been completed in 11 rounds (1989, 1991, 1993, 1997, 2000, 2004, 2006, 2009, 2011, 2015, 2018). The original survey in 1989 used a multistage, random cluster design in eight provinces to select a stratified probability sample. Further details regarding the CHNS are provided in the previous article [18].

**Figure 1:**
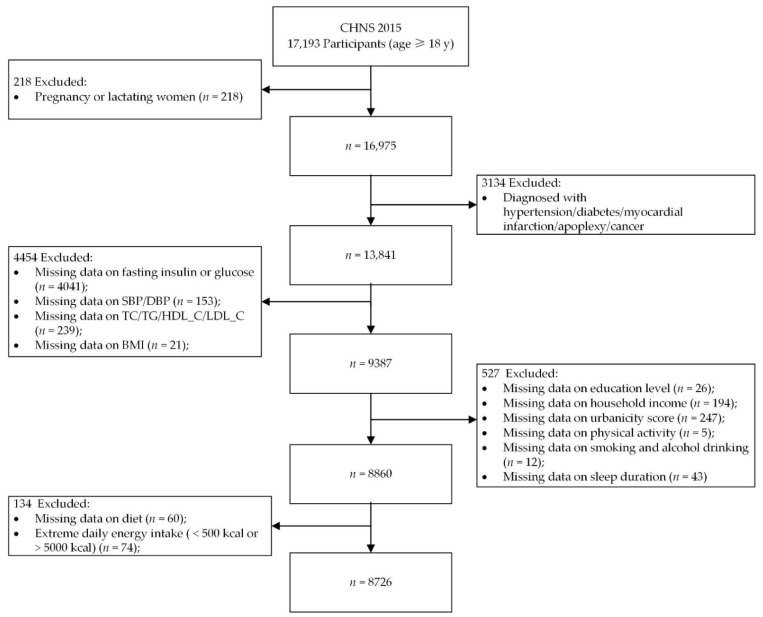
Flow chart of the included study population from the China Health and Nutrition Survey (CHNS) 2015. SBP, systolic blood pressure; DBP, diastolic blood pressure; TC, total cholesterol; TG, plasma total triglycerides; HDL_C, high-density lipoprotein cholesterol; LDL_C, low-density lipoprotein cholesterol; BMI, body mass index.

This study used data from the CHNS 2015 round. The 2015 round included 7200 households within 360 communities (60 urban neighborhoods, 60 suburban neighborhoods, 60 towns, and 180 villages) across 15 diverse provinces (Heilongjiang, Liaoning, Shandong, Henan, Hubei, Hunan, Jiangsu, Guizhou, Guangxi, Shanxi, Yunnan, Zhejiang, Chongqing, Shanghai, and Beijing). The number of adult participants (age ≥ 18 years) was 17,193. After excluding participants who were, during the period of gestation or lactation (*n* = 218), diagnosed with hypertension, diabetes, myocardial infarction, apoplexy, or cancer (*n* = 3134), with missing important laboratory measurements (*n* = 4454), with missing demographic or lifestyle information (*n* = 527), with missing data on diet (60), and with extreme daily energy intake (higher than 5000 kcal/day or lower than 500 kcal/day) (*n* = 74); a total of 8726 participants were included in the analysis (Figure 1).

### 2.2. Dietary Assessment

In the CHNS, dietary data were collected during three consecutive 24 h recalls (two weekdays and 1 weekend). Information on types and amounts of food, beverages consumed at each eating occasion (EO) during the previous 24 h were collected. There were six pre-defined EOs response options in the CHNS 24 h recall: breakfast, lunch, dinner, morning snack, afternoon snack, and evening snack, from which participants self-reported the type of each EO during the day. Energy intake at each EO was calculated by the China Food Composition and was averaged across the three days of recall to obtain mean estimates of energy intakes.

### 2.3. Definition of Meals and Snacks and Calculation of Proportion of Energy Intake from Meals, Snacks, and EOs

In the present study, breakfast, lunch, and dinner were classified as Meals. Morning snacks, afternoon snacks, and evening snacks were classified as Snacks. The proportions of energy intake from Meals and Snacks were calculated. Because energy intake from Snacks was relatively small in our sample (mean Snacks %EI = 3.0%, Supplementary Materials Table S1), in this study, we further combined breakfast and morning snack as the Morning EO, combined lunch and afternoon snack as the Noon EO, and combined dinner and evening snack as the Evening EO. The proportions of energy intake from Morning EO, Noon EO, and Evening EO were calculated and further categorized as: proportion of total energy intake = 0%; proportion of total energy intake <33.3%; proportion of total energy intake ≥33.3%.

### 2.4. Time-of-Day Energy Intake Patterns

Time-of-day energy intake patterns were determined using latent class analysis (LCA). Latent classes of time-of-day energy intake patterns were identified based on the categorization of proportion of total energy intake from Morning EO, Noon EO, and Evening EO.

### 2.5. Diet Quality

Overall diet quality was assessed by using the China Dietary Guidelines Index for Chinese adults (CDGI (2019)-A) according to Chinese Dietary Guidelines 2016. The CDGI (2019)-A has been described in detail previously [19]. Briefly, CDGI (2019)-A score is the sum of thirteen food-related components and one nutrient-related component, each scored 5 or 10 points, reflecting compliance for meeting the Chinese Dietary Guidelines 2016. The total score range is 0–110, with higher scores indicating better diet quality.

### 2.6. Blood Biochemical Measurements and Insulin Resistance Assessment

Overnight fasting blood samples were collected by trained nurses and an array of biochemical indexes were measured in a national lab in Beijing with strict quality control. Fasting plasma glucose concentration was measured by glucose oxidase-phenol and aminophenazone (GOD-PAP, Randox Laboratories Ltd., London, UK) method. Fasting insulin concentration was measured by ECL (Roche, Ltd., Basel, Switzerland). Plasma total triglycerides (TG), total cholesterol (TC), high-density lipoprotein cholesterol (HDL_C), and low-density lipoprotein cholesterol (LDL_C) were measured by cholesterol oxidase-phenol and aminophenazone (CHOD-PAP, Kyowa Medex Co., Ltd., Tokyo, Japan) method. Insulin resistance was assessed by the homeostasis model (HOMA-IR) as [fasting glucose (mmol/L) × fasting insulin (μU/mL)]/22.5 [20].

### 2.7. Anthropometric Measurements

Height, body weight, waist circumference (WC), and blood pressure (BP) were measured by trained health staff following standardized procedures. Height was measured to the nearest 0.1 cm using height tape (model 206, SECA). Body weight was measured to the nearest 0.1 kg using a body fat meter (BC601, Tanita). Body mass index (BMI) was calculated as weight (kg)/height (m)^2^. WC was measured in centimeters at the midway between the lowest rib margin and the top of the iliac crest using a SECA tape measure. BP was measured at least three times using a standard mercury sphygmomanometer after the participant resting for at least five minutes in a seated position. Systolic blood pressure (SBP) was measured at the first appearance of a pulse sound (Korotkoff phase 1) and diastolic blood pressure (DBP) was measured at the appearance of the pulse sound (Korotkoff phase 5). Mean BP of three measurements was used for analysis.

### 2.8. Covariates

Educational level was categorized into very low (completed primary school), low (completed middle school), medium (completed high school), and high (completed college). Geographic region was categorized as city, suburban, town or county, and rural village. Total physical activity including occupational, household chore, leisure time, and transportation activities was calculated into a metabolic equivalent of task (MET) per week based on the Compendium of Physical Activities [21]. Physical activity was categorized into low, middle, and high according to the tertiles of the total MET hours per week. Sleep duration was categorized into less than 6 h, 6–8 h, and more than 8 h. Smoking status was classified as non-smoker, ex-smoker, and current smoker. Alcohol drinking was classified into five groups: non-drinker, drink less than 1 time/month, drink 1–2 times/month, drink 1–4 times/week, and drink every day. Two household-level covariates are considered: Per capita household income in 2015 was calculated and categorized into low, medium, and high. Community urbanicity index was calculated based on 12 multidimensional components including physical, social, cultural, and economic environment of the community [22]. Moreover, BMI, WC, SBP, DBP, TG, TC, HDL_C, LDL_C were also considered as potential confounders.

### 2.9. Statistical Analysis

#### 2.9.1. Latent Classes of Time-of-Day Energy Intake Patterns

Latent Class Analysis (LCA) was carried out to identify distinct time-of-day energy intake patterns for adults. LCA is a method in the family of statistical approaches called finite mixture modeling, allowing identification of unobserved heterogeneity in multiple categorical response variables [23]. For this study, multiple categorical variables indicating whether or not an EO providing no energy intake, 0~33.3% or ≥33.3% of the total energy intake had occurred within Morning EO, Noon EO, and Evening EO were generated as the input variables for the LCA. A model with two latent classes was firstly tested and additional classes were added until the optimal number of latent classes was identified. The optimal latent class model was determined using information criteria-based metrics including the Akaile information criterion (AIC), Bayesian information criterion (BIC), adjusted Bayesian information criterion (aBIC), where smaller values indicate better model fit. Besides, the Lo-Mendell-Rubin Likelihood Ratio test (LMR-LRT) and the Bootstrap Likelihood ratio test (BS-LRT) were used to test whether the inclusion of an additional profile contributed to a significantly better-fitting model (*p* < 0.05 was set as the α level for nested model-fit testing). To ensure convergence on global maxima through several replications of the best log-likelihood for each model, 10,000 random sets of starting values with 50 final-stage optimizations were used. LCA was performed in SAS 9.4 (SAS Institute, Inc., Cary, NC, USA).

#### 2.9.2. Associations between Latent Classes and Sociodemographic Characteristics, Lifestyles, Eating Pattern Profiles, and Cardiometabolic Factors

Descriptive statistics for sociodemographic characteristics, lifestyles, eating pattern profiles, and cardiometabolic factors are presented. For continuous variables with normal distribution, means ± standard deviation was used. For continuous variables with non-normal distribution, median and interquartile ranges (IQR) were used. For categorical variables, number (percentages) was used. Comparison of variables across latent classes was also conducted. For continuous variables with normal distribution, the F test was used to determine differences among latent classes with Bonferroni correction to account for multiple testing. For continuous variables with non-normal distribution, Kruskal-Wallis test was used. For categorical variables, Chi-square test was used. All analyses were conducted in SAS 9.4 (SAS Institute, Inc., Cary, NC, USA). *p* < 0.05 was considered statistically significant.

#### 2.9.3. Associations between Latent Classes and Energy-Adjusted Nutrient Intakes and Diet Quality Score

General Linear Models were applied to investigate the association between latent time-of-day energy intake patterns and energy-adjusted nutrient intakes and CDGI (2019)-A score. Nutrient residual model was used for energy adjustment. General Linear Models were controlled for covariates including age, gender, education level, geographic region, smoking, alcohol drinking, physical activity, household per capita income, urbanicity index, and total energy intake. Models were checked for multicollinearity and appropriate model fit by using regression diagnostics. All analyses were conducted in SAS 9.4 (SAS Institute, Inc., Cary, NC, USA). *p* < 0.05 was considered statistically significant.

#### 2.9.4. Associations between Latent Classes and Insulin Resistance

A Multilevel Mixed-effects Logistic Regression Model was constructed to estimate the association between latent time-of-day energy intake patterns and insulin resistance estimated by HOMA-IR, taking household as the second level and individual as the first level. The upper quartile of HOMA-IR in the whole participants (HOMA-IR ≥ 2.0) was taken as the cut-off point to judge insulin resistance. Four models were conducted. Model 1 adjusted for no covariates. Model 2 adjusted for age (continuous), gender (categorical), an education level (categorical), geographic region (categorical), per capita household income (categorical), urbanicity index (continuous), physical activity (categorical), smoking (categorical), alcohol drinking (categorical), and sleep duration (categorical). Model 3 additionally adjusted for total energy intake, and CDGI (2019)-A score. Model 4 additionally adjusted for BMI, SBP, DBP, TC, TG, and HDL_C. All analyses were conducted in SAS 9.4 (SAS Institute, Inc., Cary, NC, USA). *p* < 0.05 was considered statistically significant.

## 3. Results

### 3.1. Basic Characteristics of Participants

The mean (SD) age was 50.36 (14.22) years. The numbers (percentage) of male and female participants were 3913 (44.84%) and 4813 (55.16%), respectively. Most of the participants completed middle school (34.44%), living in rural village (46.38%), had medium (35.38%) or heavy (38.07%) physical activity, had normal sleep duration (79.89%), were non-smoker (74.17%) and non-alcohol drinker (71.90%). Most of the households had low per capita household income (58.69%) (Table S1).

### 3.2. Latent Classes of Time-of-Day Energy Intake Patterns

Table 1 presents model fit statistics for LCA models estimating between one and four latent classes. Because of negative degrees of freedom in five-class solution, the five-class model was not identified, therefore, only one to four latent class models were estimated. As the number of estimated classes increased, the AIC, BIC, and aBIC generally decreased, with dramatic declines occurred at the three-class solution. All entropy remained consistently above 0.90. All the LMR-LRT indicated that the k-class model fit the data significantly better than the (k-1)-class solution (*p* < 0.001). Taken together, based on model fit tests, the goal of parsimony, and the rule of interpretability, the three-class solution was identified as the best description of latent time-of-day energy intake pattern in the present study.

**Figure 2:**
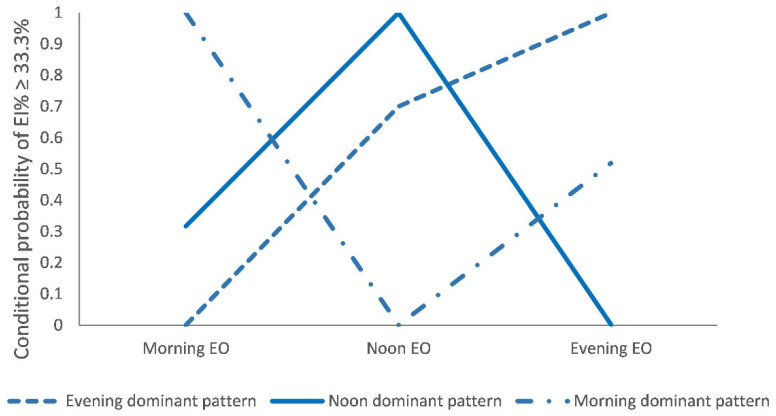
Conditional probability of proportion of total energy intake of ≥33.3% across eating occasions (EOs) in a day by latent class membership in Chinese adults. EI% ≥ 33.3% indicates energy contributed more than or equal to 33.3% total energy intake.

The time-of-day energy intake patterns were described in Figure 2. Class labels were based on distinct features of high or low conditional probability for proportion of total energy intake of ≥33.3% across three EOs of the day. The first pattern was labeled “Evening dominant pattern” (56.00%) as the probability of consuming ≥33.3% of total energy intake was relatively higher at the Evening EO than those of the Morning EO and Noon EO. The second pattern labeled “Noon dominant pattern” (28.41%) was characterized by higher conditional probabilities of consuming ≥33.3% of total energy intake at Noon EO than those of Morning EO and Evening EO. The third pattern labeled “Morning dominant pattern” (15.59%) was characterized by higher conditional probabilities of consuming ≥33.3% of total energy intake at Morning EO than those of Noon EO and Evening EO. Conditional probability of proportion of energy intake across EOs in a day for each pattern was in Figure S1.

### 3.3. Sociodemographic Characteristics, Lifestyles, and Cardiometabolic Risk Factors of Latent Classes

Table 2 presents the sociodemographic characteristics and cardiometabolic risk factors by latent time-of-day energy intake patterns. Compared with other patterns, participants in the “Evening dominant pattern” were relatively younger, had a higher proportion of males, and had more alcohol drinkers and current smokers (*p* < 0.001). Besides, participants in this pattern had a higher level of TC, TG, LDL_C, insulin, and HOMA-IR (*p* < 0.05). Participants in the “Morning dominant pattern” were older, with a lower educational level, had a higher proportion of people living in a rural area, and lower per capita household income and lower urbanicity score (*p* < 0.001).

### 3.4. Eating Pattern Profile of Latent Classes

Table 3 presents the eating pattern profiles by latent time-of-day energy intake patterns. The “Evening dominant pattern” had the lowest median energy intake proportion from Morning EO (22.64%), the “Noon dominant pattern” had the lowest median energy intake proportion from Evening EO (28.55%), and the “Morning dominant pattern” had the lowest median energy intake proportion from Noon EO (27.52%), among all the patterns (*p* < 0.001).

### 3.5. Daily Energy, Energy-Adjusted Nutrient Intakes, and Diet Quality Score of Latent Classes

Table 4 presents the daily energy, energy-adjusted nutrient intakes, and diet quality score by latent time-of-day energy intake patterns. After adjustment for multiple covariates, participants in the “Evening dominant pattern” consumed the highest daily energy intake, had the highest proportion of energy provided by total fat (%), and the highest proportion of energy provided by protein (%) (*p* < 0.001). Participants in the “Morning dominant pattern” had the highest proportion of energy provided by carbohydrates (%), and consumed the highest fiber, among all patterns (*p* < 0.001). The highest Diet Quality Score was observed in participants with “Noon dominant pattern” (*p* < 0.001).

### 3.6. Association between Latent Classes and Insulin Resistance

Table 5 presents the association between time-of-day energy intake patterns and insulin resistance (HOMA_IR ≥ 2.0). After adjusting for multi-covariates, the “Evening dominant pattern” was found to be inversely associated with insulin resistance (OR = 1.21; 95% CI = 1.05, 1.40), compared with the “Noon dominant pattern”.

## 4. Discussion

Few studies have examined time-of-day eating patterns across the day. To the best of our knowledge, studies within this regard have not been conducted in the Asian population. Using LCA, the present study identified three time-of-day energy intake patterns, “Morning dominant pattern”, “Noon dominant pattern”, and “Evening dominant pattern”, based on the proportion of total energy intake within different EOs in a Chinese adult population aged 18 years and older. Participants in the “Evening dominant pattern” were younger, had higher proportions of alcohol drinker and current smoker, consumed higher total energy intake and higher percentage of energy from fat (%), but lower percentage of energy from carbohydrate (%). The “Evening dominant pattern” was associated with higher insulin resistance risk after adjusting for multivariate covariates, compared with the “Noon dominant pattern”.

Time-of-day eating patterns can be captured by different statistical approaches depending on the dimension of the eating event that is intended to be examined [3]. The present study is aimed to find out if there is a certain kind of time-of-day pattern of energy distribution in a day that is related to health. Therefore, LCA was used to identify time-of-day energy intake patterns based on the proportions of energy intake at EOs across the day. Results showed that three latent time-of-day energy intake patterns presented in this Chinese population sample. The “Evening dominant pattern” comprised 56% of participants, demonstrating more than half of the participants had their largest energy intake in the Evening EO, including dinner and evening snacks. The second-largest pattern was the “Noon dominant pattern”, including 28.41% of participants. Participants in this pattern had their largest energy intake in the Noon EO, including lunch and afternoon snacks. The smallest pattern was the “Morning dominant pattern”, with only 15.59% of participants.

The distribution of time-of-day eating patterns found in this study was different from those identified in other populations. Among the few studies [2,3] examining time-of-day or temporal eating patterns, Eicher-Miller et al. [2] identified temporal eating patterns based on energy contribution, time of dietary intake, and number of intake occasions by using kernel k-means cluster analysis in the U.S. adult population, where four temporal dietary patterns existed. Most population (41%) belonged to the first pattern characterized by similar proportions of energy at three evenly spaced eating occasions throughout the day. The second pattern including 28% of the population represented the group having a late evening meal as the main meal of the day, while the third pattern including 22% of the population represented the group with mid-day as the main meal, and the fourth pattern including 9% of the population represented the group with sometime during noon to the evening as the main meal. The different distribution of time-of-day eating patterns among the U.S. and Chinese populations might contribute to different methodology used in studies, and also to sociocultural habits or beliefs related to eating behavior in different countries. Sociocultural and socio-economic factors that influence the time-of-day energy intake patterns need to be further studied.

The present study showed significant differences among time-of-day energy intake patterns by sociodemographic characteristics and eating pattern profiles. In the “Evening dominant pattern”, there were more males, more alcohol drinkers, and current smokers. On the contrary, there were fewer alcohol drinkers and current smokers in the “Noon dominant pattern”. It is of note that, the “Morning dominant pattern” featured lower education level, lower per capita household income, higher percentage of alcohol drinker and rural villagers in this study, which was rarely reported in previous studies.

The previous study has shown that increasing percentage of energy consumed after 5 pm or in the evening was associated with higher total energy intake [24], higher alcohol intake, and lower mean percent energy from carbohydrates [25]. In line with these findings, the present study found “Evening dominant pattern” had the highest daily energy intake and the lowest energy from carbohydrate. In view of diet quality, the present study showed the “Noon dominant pattern” had significantly higher CDGI (2019)-A score compared with the other two patterns. Different from the present study, Eicher-Miller [2] showed that the temporal dietary pattern characterized by similar proportions of energy intake during three evenly spaced eating occasions had the highest mean total Healthy Eating Index-2005 score, while the cluster characterized by mid-day (from 10:00 a.m. to 3:00 p.m.) as the largest meal in Eicher-Miller’s study had lower mean total Healthy Eating Index-2005 score. The reason may be that the relative frequency for energy intake in the morning was dramatically lower in the “mid-day pattern” in Eicher-Miller’s study, which may lead to imbalanced dietary intake and lower diet quality in this pattern. In comparison, participants in the “Noon dominant pattern” in the present study followed a healthier eating pattern, with medians of energy intake proportion from Morning EO, Noon EO, and Evening EO being 30.25%, 42.02%, and 28.55%, respectively. It is reasonable to speculate participants in this pattern may be more health-conscious and may be more willing to follow dietary recommendations.

Epidemiological studies suggest meal timing or time of energy intake has a relationship with obesity [10,14,26,27], blood pressure [12,28,29], and type 2 diabetes [10,30], but study examining time-of-day patterns of energy intake in relation to insulin resistance is lacking. The “Evening dominant pattern” observed in the present study was associated with higher insulin resistance risk, compared with the “Noon dominant pattern”, after adjusting for sociodemographic, lifestyle, and other cardiometabolic risk factors. This finding was consistent with literature reporting a positive association between energy intake at dinner and insulin resistance. In a small randomized crossover study in six healthy volunteers [31], insulin resistance was higher in the group consuming 60% of total energy in the evening than in the morning. In another weight loss intervention study [32] in 93 obese/overweight women, insulin resistance decreased in the group consuming large breakfast (~700 kcal, 50%) and small dinner (~200 kcal, 14%) compared to the group consuming small breakfast (~200 kcal, 14%) and large dinner (~700 kcal, 50%). Another experimental study showed an association between a decrease in HOMA-IR and high caloric at breakfast vs dinner in lean women with polycystic ovary syndrome [33]. A 6-year prospective cohort study showed consuming more caloric intake at dinner had a 2-fold higher incidence of diabetes at follow-up [10]. One recent cross-sectional study [17] showed that a greater proportion of energy intake in the morning was positively related to insulin sensitivity estimated by Matsuda Index. Contrary to our anticipation, no negative relationship was observed between the “Morning dominant pattern” and insulin resistance in the present study. One of the possible explanations might be that the probability of consuming ≥33.3% of total energy intake at Evening EO for this pattern (33.63%) was higher than that for the reference “Noon dominant pattern” (28.55%), which might offset the negative association between higher energy intake in the Morning EO and insulin resistance in the “Morning dominant pattern” compared to the “Noon dominant pattern”.

Circadian misalignment might explain the observed positive relationship between “Evening dominant pattern” and insulin resistance. The glucose tolerance and insulin resistance were not only robustly regulated by the central clock in the hypothalamic suprachiasmatic nucleus (SCN) but also clocks in different tissues and organs that are involved in the control of glucose metabolism. Evidence from human experimental studies [34,35,36] has demonstrated a diurnal pattern of insulin sensitivity and glucose tolerance in healthy individuals, with a higher level in the morning than in the evening. Food intake is an effective zeitgeber to regulate various peripheral body clocks and metabolic rhythms [37]. Inappropriate food intake occasion that leads to misalignment between eating behavior and tissue clock rhythms, or between central and peripheral clock rhythms, can result in circadian disruption and the development of insulin resistance [15].

Strengths of this study include using LCA as a novel approach to derive time-of-day eating patterns on the basis of the proportion of energy intake within three major EOs during the day. This person-centered approach classified participants into discrete subgroups who show similarity in energy distribution across the day. The proportion of energy intake at each EO was categorized into three categories: no energy intake, 0–33.3% energy intake, and ≥33.3% energy intake, which facilitated the identification of the largest energy intake EO across the day. Besides, the present study conducted careful screening and exclusion of participants who were diagnosed with hypertension, diabetes, myocardial infarction, apoplexy, or cancer, in case that these diseases would change eating behavior so that the actual relation would be hidden. Furthermore, the relationship between time-of-day energy intake and insulin resistance was confirmed by adjusting the physical activity, sleep duration, total energy intake, diet quality as well as cardiometabolic risk factors.

Some limitations of this study should be considered. First, LCA is a data-driven method, the time-of-day patterns derived from this study may not be generalized to other populations, and the accuracy of patterns identified by this method largely relies on the accuracy of self-reported dietary recall, which is prone to bias. Besides, because of the explanatory nature of LCA, the time-of-day energy intake patterns identified in the present study should not be viewed as indisputable. Second, meals and snacks were classified based on participant’s self-identification and time of meals and snacks was not collected in this survey so that no objective measure of EO was applied. Moreover, the day-to-day variation of meal pattern was not considered and seasonality bias could not be ruled out in the present study. Third, the excluded participants due to missing data have significant differences in variables of age, gender, and physical activity with the remaining analytical sample, indicating generalization of our results to the whole survey population should be cautious. Fourth, although we adjusted for as many covariates as possible, the possibility of other confounders unable to be included in our study could not be ruled out, such as chronotype. Lastly, causality cannot be determined and potential reverse causality bias may exist in this cross-sectional analysis.

Further studies should encompass more circadian variables in the stage of data collection, apply objective and universal meal timing definition for the sake of comparison between studies. What is more, novel statistical methods should be exploited to capture day-to-day variation and longitudinal change in time-of-day eating patterns and their relationships with health outcomes.

## 5. Conclusions

In conclusion, three distinct time-of-day energy intake patterns, “Morning dominant pattern”, “Noon dominant pattern”, and “Evening dominant pattern”, were identified in a Chinese adult population using LCA. Participants in the “Evening dominant pattern” were younger, had higher proportions of alcohol drinkers and current smokers, consumed higher daily energy intake, higher percentage of energy from fat (%), and lower percentage of energy from carbohydrate (%). Participants in the “Noon dominant pattern” had the highest diet quality score. The “Evening dominant pattern” was associated with higher insulin resistance risk after adjusting for multivariate covariates. Future research should consider more objective eating time definition, and day-to-day variation and longitudinal analysis of time-of-day eating pattern are warranted.

## Figures and Tables

**Table 1:** Model fit statistics for latent class analysis of time-of-day energy intake patterns ^1^.

Model Fit Statistics	1 Class	2 Classes	3 Classes	4 Classes
AIC	36,053.953	33,478.537	31,640.972	31,402.251
BIC	36,096.397	33,570.743	31,782.453	31,593.250
adjusted BIC	36,077.330	33,529.431	31,718.896	31,507.449
LMR-LRT	NA	<0.001	<0.001	<0.001
BS-LRT	NA	<0.001	<0.001	<0.001
Entropy	NA	1.000	0.997	0.992

^1^ AIC, Akaike Information Criterion; BIC, Bayesian Information Criterion; BS, Bootstrap; LMR, Lo-Mendell-Rubin; LRT, likelihood ratio test. NA, model fit statistic is not available.

**Table 2:** Sociodemographic characteristics, lifestyles, and cardiometabolic risk factors by latent classes.

Variables	“Evening Dominant Pattern” (*n* = 4887)	“Noon Dominant Pattern” (*n* = 2479)	“Morning Dominant Pattern” (*n* = 1360)	*p*-Value
Age (year, mean (SD))	49.27 (14.03)	51.22 (14.31)	52.72 (14.33)	<0.001
Gender (*n*, %)				
Man	2279 (46.63)	1042 (42.03)	592 (43.53)	<0.001
Woman	2608 (53.37)	1437 (57.97)	768 (56.47)	
Education level (*n*, %)				
Primary school	1301 (26.62)	749 (30.21)	460 (33.82)	<0.001
Middle school	1780 (36.42)	779 (31.42)	446 (32.79)	
High school	1109 (22.69)	567 (22.87)	294 (21.62)	
College and above	697 (14.26)	384 (15.49)	160 (11.76)	
Geographic region (*n*, %)				
City	918 (18.78)	545 (21.98)	247 (18.16)	<0.001
Suburban	946 (19.36)	382 (15.41)	165 (12.13)	
County	832 (17.02)	437 (17.63)	207 (15.22)	
Rural village	2191 (44.83)	1115 (44.98)	741 (54.49)	
Physical activity (*n*, %)				
Low	1253 (25.64)	677 (27.31)	387 (28.46)	0.077
Middle	1744 (35.69)	894 (36.06)	449 (33.01)	
High	1890 (38.67)	908 (36.63)	524 (38.53)	
Sleep duration (*n*, %)				
6–8 h	3940 (80.62)	1968 (79.39)	1063 (78.16)	0.063
<6 h	110 (2.25)	77 (3.11)	33 (2.43)	
>8 h	837 (17.13)	434 (17.51)	264 (19.41)	
Smoking (*n*, %)				
Nonsmoker	3536 (72.36)	1918 (77.37)	1018 (74.85)	<0.001
Ex-smoker	111 (2.27)	46 (1.86)	32 (2.35)	
Current smoker	1240 (25.37)	515 (20.77)	310 (22.79)	
Alcohol drinking (*n*, %)				
Nondrinker	3414 (69.86)	1845 (74.43)	1015 (74.63)	<0.001
Drink ≤1 time/month	273 (5.59)	128 (5.16)	59 (4.34)	
Drink 1–2 times/month	355 (7.26)	139 (5.61)	66 (4.85)	
Drink 1–4 times/week	459 (9.39)	221 (8.91)	112 (8.24)	
Drink everyday	386 (7.90)	146 (5.89)	108 (7.94)	
Per capita household income (*n*, %)				
Low	2925 (59.85)	1455 (58.69)	872 (64.12)	0.007
Medium	1828 (37.41)	959 (38.68)	465 (34.19)	
High	134 (2.74)	65 (2.62)	23 (1.69)	
Urbanicity score (mean (SD))	72.70 (17.06)	71.29 (18.23)	69.58 (17.80)	<0.001
BMI (mg/kg^2^, mean (SD))	23.70 (3.57)	23.94 (3.74)	23.79 (3.53)	0.029
SBP (mmHg, mean (SD))	123.84 (16.50)	124.30 (17.45)	125.03 (17.23)	0.063
DBP (mmHg, mean (SD))	79.57 (10.38)	79.56 (10.26)	80.13 (10.30)	0.179
TC (mmol/L, mean (SD))	4.94 (1.06)	4.84 (1.06)	4.82 (0.97)	<0.001
TG (log mmol/L, mean (SD)) ^1^	0.18 (0.59)	0.13 (0.57)	0.17 (0.55)	0.010
LDL_C (mmol/L, mean (SD))	3.12 (0.89)	3.06 (0.92)	3.04 (0.85)	0.002
HDL_C (mmol/L, mean (SD))	1.29 (0.33)	1.28 (0.34)	1.29 (0.32)	0.866
Glucose (log mmol/L, mean (SD)) ^1^	1.65 (0.18)	1.65 (0.18)	1.65 (0.19)	0.637
Insulin (log μU/mL, mean (SD)) ^1^	1.79 (0.64)	1.74 (0.62)	1.74 (0.66)	0.003
HOMA-IR (log mean (SD)) ^1^	0.32 (0.71)	0.28 (0.69)	0.27 (0.72)	0.018

^1^ Log-transformation was conducted to improve normality. SD, standard deviation; BMI, body mass index; SBP, systolic blood pressure; DBP, diastolic blood pressure; TC, total cholesterol; TG, plasma total triglycerides; HDL_C, high-density lipoprotein cholesterol; LDL_C, low-density lipoprotein cholesterol; HOMA-IR, insulin resistance assessed by homeostasis model.

**Table 3:** Eating pattern profile by latent classes ^1^.

Eating Pattern Profile	“Evening Dominant Pattern” (*n* = 4887)	“Noon Dominant Pattern” (*n* = 2479)	“Morning Dominant Pattern” (*n* = 1360)	*p*-Value ^2^
EI from Morning EO (%) ^3^	22.64 (17.75, 27.04)	30.25 (25.25, 34.39)	39.39 (36.27, 44.08)	<0.001
EI from Noon EO (%)	36.60 (32.33, 40.95)	42.02 (37.92, 46.79)	27.52 (22.69, 30.43)	<0.001
EI from Evening EO (%)	40.49 (36.96, 45.05)	28.55 (24.59, 31.09)	33.63 (29.75, 38.16)	<0.001

^1^ Values are median (P25, P75). ^2^ Kruskal-Wallis test comparison among latent classes (*p* < 0.05). ^3^ EI, energy intake.

**Table 4:** Daily energy, energy-adjusted nutrient intakes, and diet quality score by latent classes.

Variables	“Evening Dominant Pattern” (*n* = 4887)	“Noon Dominant Pattern” (*n* = 2479)	“Morning Dominant Pattern” (*n* = 1360)	*p*-Value
Energy and Nutrient Intakes ^1^				
Energy, kcal	2094.63 (10.06) ^a^	1997.50 (14.10) ^b^	1895.06 (19.07) ^c^	<0.001
Carbohydrate, g	241.16 (0.99) ^c^	257.52 (1.39) ^b^	271.70 (1.88) ^a^	<0.001
Carbohydrate, EI%	48.04 (0.17) ^c^	51.32 (0.24) ^b^	53.48 (0.33) ^a^	<0.001
Total fat, g	84.82 (0.44) ^a^	79.70 (0.61) ^b^	74.34 (0.83) ^c^	<0.001
Total fat, EI%	37.29 (0.17) ^a^	34.96 (0.24) ^b^	33.29 (0.32) ^c^	<0.001
Protein, g	70.16 (0.27) ^a^	66.90 (0.38) ^b^	65.00 (0.51) ^c^	<0.001
Protein, EI%	14.02 (0.05) ^a^	13.35 (0.07) ^b^	12.88 (0.10) ^c^	<0.001
Fiber, g	12.46 (0.11) ^b^	12.55 (0.16) ^a,b^	13.15 (0.21) ^a^	0.016
Vitamin C, mg	93.00 (2.20) ^a^	82.33 (3.09) ^b^	81.70 (4.19) ^b^	0.005
Calcium, mg	375.88 (2.76)	369.57 (3.86)	369.50 (5.24)	0.323
Iron, mg	22.14 (0.14)	22.40 (0.19)	21.87 (0.26)	0.255
Zinc, mg	11.02 (0.04) ^a^	10.41 (0.06) ^b^	10.46 (0.07) ^b^	<0.001
Sodium, mg	4659.45 (100.10)	4835.83 (140.18)	5083.83 (190.06)	0.129
Potassium, mg	1711.98 (8.87) ^a^	1664.58 (12.42) ^b^	1658.83 (16.85) ^b^	0.001
Phosphorus, mg	966.00 (3.19)	959.28 (4.47)	961.88 (6.06)	0.461
Diet Quality Score ^2^				
CDGI (2019)-A score	50.93 (0.14) ^b^	51.72 (0.20) ^a^	50.67 (0.27) ^b^	<0.001

^1^ Values are means (standard error) adjusted for age, gender, education level, geographic region, smoking, alcohol drinking, physical activity, household per capita income, urbanicity index, and total energy intake. Different superscript letters indicate significant *t*-test pairwise comparisons of the mean, with adjustment for multiple comparisons, between latent classes (*p* < 0.05). Nutrient intakes were adjusted for total energy intake by the nutrient residual model. Different superscript letters indicate significant *t*-test pairwise comparisons, with adjustment for multiple comparisons, between latent classes (*p* < 0.05). ^2^ CDGI (2019)-A, China Dietary Guidelines Index for Chinese adults according to Chinese Dietary Guidelines 2016. Means (standard error) adjusted for age, gender, education level, geographic region, smoking, alcohol drinking, physical activity, household per capita income, urbanicity index, and total energy intake. Different superscript letters (^a^, ^b^, ^c^) indicate significant *t*-test pairwise comparisons, with adjustment for multiple comparisons, between latent classes (*p* < 0.0167). ^a^, ^b^, ^c^ represent latent classes with the highest, medium, respectively, lowest mean values.

**Table 5:** Association between time-of-day energy intake patterns and insulin resistance (OR, 95% CI) ^1^.

Models	“Noon Dominant Pattern” (*n* = 2479)	“Evening Dominant Pattern” (*n* = 4887)	“Morning Dominant Pattern” (*n* = 1360)
Model 1	1	1.16(1.02–1.32) *	1.01(0.85–1.20)
Model 2	1	1.15(1.01–1.31) *	1.05(0.88–1.25)
Model 3	1	1.14(1.00–1.30) *	1.05(0.88–1.26)
Model 4	1	1.21(1.05–1.40) *	1.08(0.89–1.31)

^1^ A Multilevel Mixed-effects Logistic Regression Model was constructed to estimate the association between latent time-of-day energy intake patterns and insulin resistance (binary, HOMA-IR ≥ 2.0 as the cutoff), taking household as the second level and individual as the first level. Model 1 adjusted for no covariates. Model 2 adjusted for age (continuous), gender (categorical), education level (categorical), geographic region (categorical), per capita household income (categorical), urbanicity index (continuous), physical activity (categorical), smoking (categorical), alcohol drinking (categorical), and sleep duration (categorical). Model 3 additionally adjusted for total energy intake, and CDGI (2019)-A score. Model 4 additionally adjusted for BMI, SBP, DBP, TC, TG, HDL_C. * *p* < 0.05.

## Data Availability

No new data were created or analyzed in this study. Data sharing is not applicable to this article.

## Supplementary Materials

The following are available online at https://www.mdpi.com/2072-6643/13/3/725/s1, Table S1: Sociodemographic characteristics, lifestyles, eating pattern profile, and cardiometabolic risk factors of the overall participants; Figure S1: Conditional probability of proportion of energy intake across EOs in a day by latent class membership in Chinese adults.

## References

[1] Almoosawi S., Vingeliene S., Gachon F., Voortman T., Palla L., Johnston J.D., Van Dam R.M., Darimont C., Karagounis L.G. (2019). Chronotype: Implications for Epidemiologic Studies on Chrono-Nutrition and Cardiometabolic Health. Adv. Nutr..

[2] Eicher-Miller H.A., Khanna N., Boushey C.J., Gelfand S.B., Delp E.J. (2016). Temporal Dietary Patterns Derived among the Adult Participants of the National Health and Nutrition Examination Survey 1999–2004 Are Associated with Diet Quality. J. Acad. Nutr. Diet..

[3] Leech R., Worsley A., Timperio A., McNaughton S. (2017). Temporal eating patterns: A latent class analysis approach. Int. J. Behav. Nutr. Phys. Act..

[4] Stenvers D.J., Jonkers C.F., Fliers E., Bisschop P., Kalsbeek A. (2012). Nutrition and the circadian timing system. Prog. Brain Res..

[5] Bi H., Gan Y., Yang C., Chen Y., Tong X., Lu Z. (2015). Breakfast skipping and the risk of type 2 diabetes: A meta-analysis of observational studies. Public Health Nutr..

[6] Ofori-Asenso R., Owen A.J., Liew D. (2019). Skipping Breakfast and the Risk of Cardiovascular Disease and Death: A Systematic Review of Prospective Cohort Studies in Primary Prevention Settings. J. Cardiovasc. Dev. Dis..

[7] Chen H.J., Chuang S.Y., Chang H.Y., Pan W.H. (2019). Energy intake at different times of the day: Its association with elevated total and LDL cholesterol levels. Nutr. Metab. Cardiovasc. Dis..

[8] Nakajima K., Suwa K. (2015). Association of hyperglycemia in a general Japanese population with late-night-dinner eating alone, but not breakfast skipping alone. J. Diabetes Metab. Disord..

[9] Aljuraiban G.S., Chan Q., Griep L.M.O., Brown I.J., Daviglus M.L., Stamler J., Horn L.V., Paul Elliott M., Frost G.S. (2015). The impact of eating frequency and time of intake on nutrient quality and body mass index: The INTERMAP Study, a population based study. J. Acad. Nutr. Diet..

[10] Bo S., Musso G., Beccuti G., Fadda M., Fedele D., Gambino R., Gentile L., Durazzo M., Ghigo E., Cassader M. (2014). Consuming More of Daily Caloric Intake at Dinner Predisposes to Obesity. A 6-Year Population-Based Prospective Cohort Study. PloS ONE.

[11] Leech R.M., Livingstone K.M., Worsley A., Timperio A., McNaughton S.A. (2016). Meal Frequency but Not Snack Frequency Is Associated with Micronutrient Intakes and Overall Diet Quality in Australian Men and Women. J. Nutr..

[12] Leech R.M., Timperio A., Worsley A., McNaughton S.A. (2019). Eating patterns of Australian adults: Associations with blood pressure and hypertension prevalence. Eur. J. Nutr..

[13] Khanna N., Eicher-Miller H.A., Boushey C.J., Gelfand S.B., Delp E.J. Temporal Dietary Patterns Using Kernel k-Means Clustering. Proceedings of the 2011 IEEE International Symposium on Multimedia.

[14] Aqeel M.M., Guo J., Lin L., Gelfand S.B., Delp E.J., Bhadra A., Richards E.A., Hennessy E., Eicher-Miller H.A. (2020). Temporal Dietary Patterns Are Associated with Obesity in US Adults. J. Nutr..

[15] Stenvers D.J., Scheer F., Schrauwen P., la Fleur S.E., Kalsbeek A. (2019). Circadian clocks and insulin resistance. Nat. Rev. Endocrinol..

[16] Wefers J., van Moorsel D., Hansen J., Connell N.J., Havekes B., Hoeks J., van Marken Lichtenbelt W.D., Duez H., Phielix E., Kalsbeek A. (2018). Circadian misalignment induces fatty acid metabolism gene profiles and compromises insulin sensitivity in human skeletal muscle. Proc. Natl. Acad. Sci. USA.

[17] Rangaraj V.R., Siddula A., Burgess H.J., Pannain S., Knutson K.L. (2020). Association between Timing of Energy Intake and Insulin Sensitivity: A Cross-Sectional Study. Nutrients.

[18] Zhang B., Zhai F.Y., Du S.F., Popkin B.M. (2014). The China Health and Nutrition Survey, 1989–2011. Obes. Rev..

[19] Huang F., Wang Z., Wang L., Wang H., Zhang J., Du W., Su C., Jia X., Ouyang Y., Wang Y. (2019). Evaluating adherence to recommended diets in adults 1991–2015: Revised China dietary guidelines index. Nutr. J..

[20] Matthews D.R., Hosker J.P., Rudenski A.S., Naylor B.A., Treacher D.F., Turner R.C. (1985). Homeostasis model assessment: Insulin resistance and beta-cell function from fasting plasma glucose and insulin concentrations in man. Diabetologia.

[21] Ainsworth B.E., Haskell W.L., Whitt M.C., Irwin M.L., Swartz A.M., Strath S.J., O’Brien W.L., Bassett D.R., Schmitz K.H., Emplaincourt P.O. (2000). Compendium of physical activities: An update of activity codes and MET intensities. Med. Sci. Sports Exerc..

[22] Su C., Song X., Hu H., Du W., Wang H., Zhang B. (2020). Longitudinal Association between Urbanicity and Total Dietary Fat Intake in Adults in Urbanizing China from 1991 to 2015: Findings from the CHNS. Nutrients.

[23] Collins L.M., Lanza S.T. (2010). Latent Class and Latent Transition Analysis with Applications in the Social, Behavioral and Health Sciences.

[24] De Castro J.M. (2004). The time of day of food intake influences overall intake in humans. J. Nutr..

[25] Kant A.K., Schatzkin A., Ballard-Barbash R. (1997). Evening eating and subsequent long-term weight change in a national cohort. Int. J. Obes..

[26] Wang J.B., Patterson R.E., Ang A., Emond J.A., Shetty N., Arab L. (2014). Timing of energy intake during the day is associated with the risk of obesity in adults. J. Hum. Nutr. Diet..

[27] Leech R.M., Timperio A., Livingstone K.M., Worsley A., McNaughton S.A. (2017). Temporal eating patterns: Associations with nutrient intakes, diet quality, and measures of adiposity. Am. J. Clin. Nutr..

[28] Keller K., Rodriguez Lopez S., Carmenate Moreno M. (2017). Association between meal intake behavior and blood pressure in Spanish adults. Nutr. Hosp..

[29] Almoosawi S., Prynne C.J., Hardy R., Stephen A.M. (2013). Time-of-day of energy intake. J. Hypertens..

[30] Beccuti G., Monagheddu C., Evangelista A., Ciccone G., Broglio F., Soldati L., Bo S. (2017). Timing of food intake: Sounding the alarm about metabolic impairments? A systematic review. Pharmacol. Res..

[31] Morgan L.M., Shi J.W., Hampton S.M., Frost G. (2012). Effect of meal timing and glycaemic index on glucose control and insulin secretion in healthy volunteers. Br. J. Nutr..

[32] Jakubowicz D., Barnea M., Wainstein J., Froy O. (2013). High caloric intake at breakfast vs. dinner differentially influences weight loss of overweight and obese women. Obesity.

[33] Jakubowicz D., Barnea M., Wainstein J., Froy O. (2013). Effects of caloric intake timing on insulin resistance and hyperandrogenism in lean women with polycystic ovary syndrome. Clin. Sci..

[34] Morris C.J., Yang J.N., Garcia J.I., Myers S., Bozzi I., Wang W., Buxton O.M., Shea S.A., Scheer F.A. (2015). Endogenous circadian system and circadian misalignment impact glucose tolerance via separate mechanisms in humans. Proc. Natl. Acad. Sci. USA.

[35] Gibson T., Jarrett R.J. (1972). Diurnal variation in insulin sensitivity. Lancet.

[36] Saad A., Dalla Man C., Nandy D.K., Levine J.A., Bharucha A.E., Rizza R.A., Basu R., Carter R.E., Cobelli C., Kudva Y.C. (2012). Diurnal pattern to insulin secretion and insulin action in healthy individuals. Diabetes.

[37] Patton D.F., Mistlberger R.E. (2013). Circadian adaptations to meal timing: Neuroendocrine mechanisms. Front. Neurosci..

