# Supplementary material for: Association of Time-of-Day Energy Intake Patterns with Nutrient Intakes, Diet Quality, and Insulin Resistance
Source: Nutrients. 2021 Feb 25;13(3):725. doi: 10.3390/nu13030725 (PMC7996289; doi:10.3390/nu13030725)
Supplement: Supplementary file 1 [file nutrients-13-00725-s001.pdf]

**Supplementary Materials:**

**Table S1**: Sociodemographic characteristics, lifestyles, eating pattern profile, and cardiometabolic risk factors of the overall participants

| Variables | Mean (SD) % |
| --- | --- |
| Sociodemographic characteristics and lifestyles | |
| Age (y, mean (SD)) | 50.36(14.22) |
| Gender | |
| Man | 3913（44.84） |
| Woman | 4813（55.16) |
| Education level (%) | |
| Very low | 2510（28.76） |
| Low | 3005（34.44） |
| Medium | 1970（22.58） |
| High | 1241（14.22） |
| Geographic region (%) | |
| City | 1710（19.60） |
| Suburban | 1493（17.11） |
| Town or county | 1476（16.91） |
| Rural village | 4047（46.38） |
| Physical activity (%) | |
| Light | 2317（26.55） |
| Medium | 3087（35.38） |
| Heavy | 3322（38.07） |
| Sleep duration (%) | |
| Normal | 6971(79.89) |
| Short | 220(2.52) |
| Long | 1535(17.59) |
| Smoking (%) | |
| Nonsmoker | 6472（74.17） |
| Ex-smoker | 189（2.17） |
| Current smoker | 2065（23.66） |
| Alcohol drinking (%) | |
| Nondrinker | 6274（71.90） |
| Drink less than 1 time/month | 460（5.27） |
| Drink 1-2 times/month | 560（6.42） |
| Drink 1-4 times/week | 792（9.08） |
| Drink everyday | 640（7.33） |
| Per capita household income | |
| Low | 5252（60.19） |
| Medium | 3252（37.27） |
| High | 222（2.54） |
| Urbanicity (mean (SD)) | 71.81(17.55) |

| | |
|---|---|
| Eating pattern profile | |
| EI% from Meals (mean (SD)) | 96.98(5.52) |
| EI% from Snacks (mean (SD)) | 3.02(5.52) |
| Cardiometabolic risk factors | |
| BMI (mg/kg, mean (SD)) | 23.78(3.61) |
| SBP (mmHg, mean (SD)) | 124.15(16.89) |
| DBP (mmHg, mean (SD)) | 79.65(10.33) |
| TC (mmol/L, mean (SD)) | 4.89(1.05) |
| TG (log mmol/L, mean (SD)) | 0.16(0.58) |
| LDL_C (mmol/L, mean (SD)) | 3.09(0.89) |
| HDL_C (mmol/L, mean (SD)) | 1.29(0.33) |
| Glucose (log mmol/L, mean (SD)) [1] | 1.65(0.18) |
| Insulin (log μU/mL, mean (SD)) [1] | 1.77(0.64) |
| HOMA-IR (log mean (SD)) [1] | 0.30(0.71) |

[1] Log-transformation was conducted to improve normality. SD, standard deviation; BMI, body mass index; SBP, systolic blood pressure; DBP, diastolic blood pressure; TC, total cholesterol; TG, plasma total triglycerides; HDL_C, high-density lipoprotein cholesterol; LDL_C, low-density lipoprotein cholesterol; HOMA-IR, insulin resistance assessed by homeostasis model.

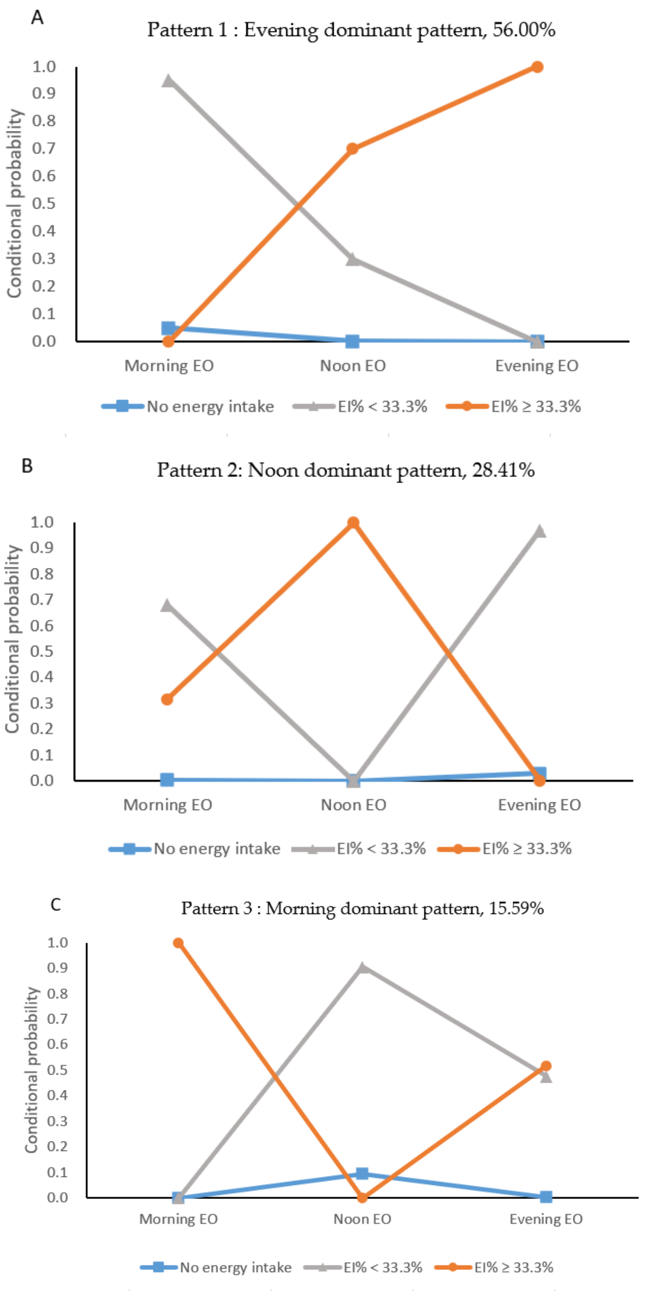


**Figure 1.** Conditional probability of proportion of energy intake across EOs in a day by latent class membership in Chinese adults. (A) "Noon dominant pattern"; (B) "Evening dominant pattern"; (C) "Morning dominant pattern". EI% < 33.3% indicates energy contributed less than 33.3% total energy intake; EI% ≥ 33.3% indicates energy contributed more than or equal to 33.3% total energy intake.
